# Rehab-AMD: co-design of an application for visual rehabilitation and monitoring of Age-related Macular Degeneration

**DOI:** 10.1186/s12911-024-02625-w

**Published:** 2024-08-23

**Authors:** Guadalupe González-Montero, María Guijarro Mata-García, Carlos Moreno Martínez, Joaquín Recas Piorno

**Affiliations:** 1 Department Optometría y Visión, Complutense University of Madrid, C. de Arcos de Jalón, 118, Madrid, 28037 Spain; 2 Department of Computer Architecture and Automation, Complutense University of Madrid, Calle del Prof. José García Santesmases, 9, Madrid, 28040 Spain; 3 Department de Ciencia, Computación y Tecnología, Universidad Europea, C. Tajo, Villaviciosa de Odón, 28670 Madrid Spain

**Keywords:** Co-design, Vision rehabilitators, Mobile application, Technological solution, Artificial intelligence

## Abstract

**Background:**

The increasing demand for remote medical care, driven by digital healthcare advancements and the COVID-19 pandemic, necessitates effective solutions tailored to patients and healthcare practitioners. Co-design, involving collaboration between software developers, patients, and healthcare practitioners, prioritizes end-user needs. Research indicates that integrating patient perspectives enhances user experience and usability. However, its application in healthcare has been limited to small projects. This work focuses on co-designing a technological solution to enhance the monitoring and visual rehabilitation of individuals with Age-Related Macular Degeneration (AMD), a condition that significantly impacts the quality of life in people over 60. Current vision rehabilitation systems lack personalization, motivation, and effective progress monitoring. Involving patients and healthcare practitioners in the design process aims to ensure the final product meets their needs.

**Methods:**

The project employs iterative and collaborative principles, involving a vision rehabilitation expert and two AMD patients as active users in the application’s development and validation. The process begins by establishing requirements for user accounts and rehabilitation exercises. It continues with an initial approach extended through user validation. Co-design is facilitated by specific workshops marking each project iteration, totaling four workshops, along with continuous communication sessions between experts and developers to validate design decisions. Initial requirements gathering and constant feedback from end-users, the visual rehabilitator, and patients are crucial for refining the product effectively.

**Results:**

The workshops produced a prototype featuring a test to monitor changes and progression and 15 visual rehabilitation exercises. Numerous patient and vision rehabilitation-driven software modifications led to a final design that is responsive and adaptive to end-user needs.

**Conclusions:**

The Rehab-AMD pilot project aims to develop a collaborative and adaptive software solution for AMD rehabilitation by actively involving stakeholders and applying iterative design principles. Co-design in the Rehab-AMD solution proves to be a methodology that identifies usability issues and needs from the initial design stages. This approach ensures that software developers create a final product that is genuinely useful and manageable for people with AMD and the targeted vision rehabilitators.

## Background

The need for remote medical care and monitoring demanded by today’s society has been driven by new technologies and the COVID-19 pandemic [1, 2]. For these services to be effective, it is important that they are tailored to the needs of patients and healthcare practitioners [3] and use collaborative approaches such as co-design [4].

To ensure that these new digital healthcare applications meet the needs of patients, it is critical to involve them in the design and development process. Co-design is a principle in which the community are treated as equal collaborators in a design process to ensure that they are usable and adaptable [5]. Although there are increasing advocates of this methodology in health care [4], there is little evidence on its effectiveness in different patient groups [6].

For these reasons, this research work focuses on the co-design of a healthcare methodology for patients with Age-related Macular Degeneration (AMD). AMD is a disease of the retina and the most common cause of irreversible central vision loss in people over 60 years of age [7]. It is estimated that nearly 300 million people worldwide will suffer from AMD by 2040 [8]. The progressive reduction in visual acuity affects daily life tasks such as driving, reading, operating a cell phone, or recognizing faces and generates significant functional loss [9], reducing quality of life and causing depression [10].

Early detection is essential to apply the different therapeutic options as soon as possible and slow down the progression [11]. Visual rehabilitation, which consists of learning to see with the undamaged areas of the retina, is the best option to cope with vision loss [12] and to have a lower impact on quality of life and mood. In the case of AMD, the goal is to locate the most functional peripheral area or best Preferred Retinal Locus (PRL) [13] to train it.

Currently, microperimetry makes it possible to locate and train the PRL [14], however, rehabilitation sessions with the necessary apparatus must be carried out in the consultation room and with the practitioner who must prescribe exercises to be performed at home. These exercises are usually not very motivating, as they are defined in a generic way for all patients, and not specifically adapted to the specific vision loss nor do they allow adjusting the degree of difficulty, resulting either too easy or too difficult. These are exercises to be performed on a paper template or by looking at everyday objects in the patient’s environment that do not change or adapt and base rehabilitation progress on repetition. In addition, these types of exercises do not allow monitoring of the patient’s progress, so that no feedback can be given to the user, information that we consider key to motivate the patient to continue with the rehabilitation as indicated by some models of behavioural change and motivation used in other therapies [15, 16]. At the same time, it is worth highlighting the importance of the patients’ mood and how periodic monitoring over time would ensure therapeutic compliance [17], but this is not possible due to the current system of exercise design.

For all these reasons, the co-design of software presents itself as a valuable opportunity in the field of healthcare for patients with AMD, as it allows the patient to be introduced into the design phase with the aim that his or her perspective during the development process will serve to create a suitable tool that allows training and monitoring to be carried out comfortably and effectively. Nowadays, there is a growing interest and examples of the use of agile models and co-design for the development of software applications in the healthcare sector, which require a tailored approach [18], although they are currently limited to small-scale projects [19]. In these projects, co-design is used to improve solutions for patients, caregivers and healthcare practitioners [20], with a focus on involving end users and understanding their specific needs. Co-design is a creative process in which software developers and people who do not have programming expertise collaborate to find innovative solutions [21].

In the context of software development, co-design involves collaboration between developers and people without experience, paying particular attention to the needs and opinions of end users [22, 23]. The literature suggests that integrating patient’s vision into software design is feasible and can improve usability and user experience [24].

The agile software development process focuses on collaboration with users and rapid software deployment [25]. However, if testing is to be extended to a mobile device, periodic end-user input and reviews are needed, as direct translation between devices may not be adequate. In these types of projects, where requirements may not be well defined at the beginning and may emerge over time, agile methodology seems to be most appropriate [26]. Specifically, in the case of complex healthcare technology development, iterative co-design workshops are essential to ensure that the final product fits the needs of patients, based on the experience of experts through periodic workshops.

Intelligent recommender systems are tools that provide effective recommendations on what actions users can take or what information they can consume [27, 28]. They are effective in performing decision support tasks based on user preferences [29–31]. There are many approaches to recommender systems, such as collaborative, content filtering-based, knowledge-based, utility-based, ontology-based, demographic-based, and hybrid recommender systems [32]. Medical recommender systems use the methods and algorithms of medical information recommender systems [33, 34].

The objective, therefore, of this pilot project is the co-design of an app accessible through an agile-based methodology, Rehab-AMD. This tool allows remote rehabilitation, under supervision of the online visual rehabilitator, adapting to the patient’s needs, learning from progress and deficiencies, and being able to record the patient’s progress, sending regular patient information to the visual rehabilitator to monitor the adaptation proposed by the AI of the application.

## Methods

A pilot project is proposed as a co-design of the solution, Rehab-AMD, to evaluate the suitability of a software product designed for the visual rehabilitation, monitoring and information management of AMD patients. The pilot project applies a highly collaborative working model between stakeholders, such as AMD visual rehabilitators (as domain experts), representatives of AMD patient groups, and technical teams (software developers).

This approach is motivated by three elements of the initial context. To begin with, the current system is based on complex and expensive devices that require the presence of a practitioner to operate them [35, 36]. In turn, rehabilitation exercises are designed on paper and to be performed outside the visual rehabilitator’s office [37, 38] following rehabilitation techniques that are not standardized and present great heterogeneity [39], making it very difficult for the patient to perform them correctly. Another fundamental aspect is the high complexity of the process to be implemented in the system, highly dependent on the knowledge and experience of the subject matter expert, as well as on the specific evolution of each patient. The third factor is the need to produce a design adaptable to the changing circumstances of the patient during the use of the system. These factors motivate the application of the principles for inclusive software development of the RiD framework [40], and its materialization through the working dynamics of agile development models, specifically inspired in SCRUM, which facilitate continuous feedback and high cooperation in the appropriate design with this starting context [41]. Co-design requires applying iterative and collaborative design principles between subject matter expert and technical team to define requirements, validation, and acceptance testing, according to the study by [20].

The model used in the pilot project is inspired by these principles and is therefore based on capturing the functional needs of the users, where the protagonists are the domain experts (visual rehabilitators) and the representatives of AMD patients, as users of the future application. Likewise, the model includes the active participation of users in the iterative development and validation with direct involvement, providing feedback in each iteration as a fundamental axis of the co-design.

**Fig. 1:**
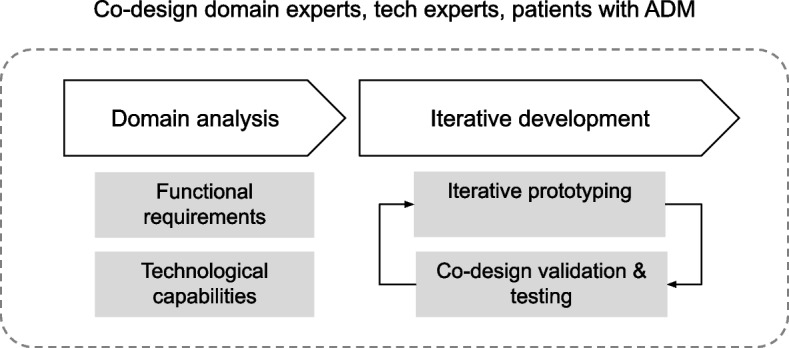
Collaborative project model applied to co-design of Rehab-AMD application

The pilot project therefore starts with the development of basic functionalities, increasing the functionality as the result and the validation by the users during the project progresses. The model applied to the pilot project based on iterative design and development is shown in Fig. 1.

The co-design of digital healthcare applications in the healthcare sector benefits from the realization of working sessions (Workshops, WKS) with representatives of patients and healthcare specialists. The process has a fundamental basis in extracting knowledge and opinion from experts and users, within a multidisciplinary composition of the team. The focus of the work meetings is inspired by the Design Thinking process for insight gathering [42]. Specific activities are also applied to capture contextual analysis of both profiles in the context of their work as recommended by [43]. In this sense, the pilot project is structured around the execution of specific workshops that mark the beginning of each iteration in the project plan, with a total of 4 workshops, preceded by an initial planning session and identification of participants. Between each workshop, communication between domain experts and developers is maintained in a flexible and continuous manner for the validation of design decisions, through short face-to-face meetings, online or written communication (email). In essence, the requirements capture is initial, and the progress throughout the pilot project consists of capturing feedback from both end-user profiles, visual rehabilitators, and patients, for the adjustment of the product.

**Fig. 2:**
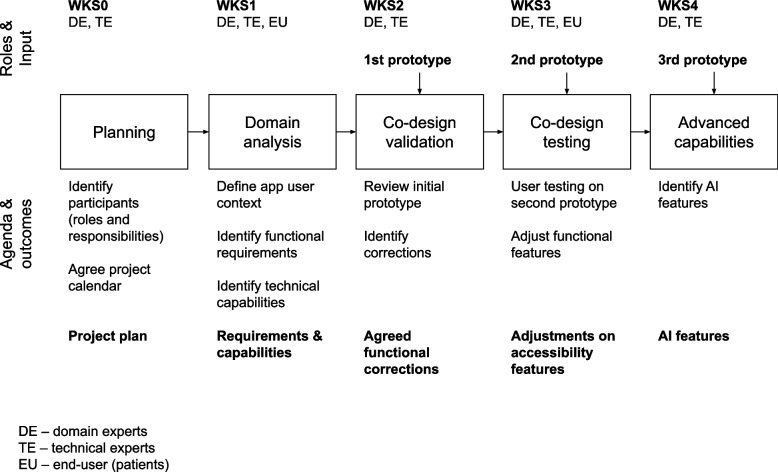
Methodology: Workshop structure and iterative co-design in pilot project

Figure 2 summarizes the iterative co-design structure, showing the collaborative workshops, the roles involved, and the results obtained in each of them. Each iteration and the corresponding workshops are described below.

### WKS0: initial planning

In an initial meeting, which corresponds to WKS0 in Fig. 2, the general starting point (user context) was analyzed based on the testimony of the domain experts. In this same meeting, the planning of the pilot project was defined, resulting in the identification of participants and their roles in the project, the general work plan and schedule, as well as a proposal for the agendas of each workshop. The design team consisted of three technical experts, a vision rehabilitation specialist and two patients (one male and one female) diagnosed with AMD in the early stages of the disease (aged 65 and 59 years respectively). The participating patients had to be people who had some use of systems such as mobile phones and tablets and had to be in the early stages of the disease, as this is where vision rehabilitation is generally applied. Both were following classical rehabilitation methods and were keen to try alternative systems.

The work structures and teams chosen have a cross-functional approach, depending on the agenda of each meeting. The profiles involved in the pilot, and their role in the project are shown in Table 1.

**Table 1:** Profiles and their role in the collaborative project

Profile	Project role	Comments
Visual rehabilitation expert	Domain expert	1 people (advance expertise level in visual rehabilitation)
	Product owner	
Software Engineer	Development team	4 people (Expertise in: mobile app design, databases, mockup and user interface, SW development)
Coordinator	Development coordination	2 people (project management expertise)
	Main contact for domain expert	
Patient with AMD	End user	2 people (male and female, 65 and 59 years old, early AMD diagnosis)
	Beta-testers	

### WKS1: requirements capture and domain analysis

In this first design meeting, the needs to be covered by the application are identified, with input from domain experts, AMD patients and the collaboration of the developers. The work extends to several interactions and verification over four weeks, to complete the understanding of the functional requirements and the examples provided by the visual rehabilitators. The domain expert provides paper-based ideas of graphics for screen layouts and visual exercises. Paper-based drafts of the visualization and type of exercises expected in the application are jointly elaborated as a result of the working sessions, together with the list of functional objectives of the application.

### WKS2: validation of the co-design

In the second workshop, development technicians present a first prototype based on the functionalities identified in the previous phase, and it is reviewed jointly with the domain experts. This ensures that the initial specifications are correctly captured in the prototype, and the list of features to be added in the next iteration are analyzed and prioritized.

Aspects to be reviewed focus on user interaction with the application, data entry, the graphical design of the Amsler grid [44], and initial exercises to be considered in the design. This iteration lasts approximately 2 weeks.

### WKS3: user testing

This workshop is attended by domain experts, developers, and AMD patient representatives. A joint review of the prototype developed to date is carried out to show the prototype and explain how to use the application to AMD patients.

At the end of the phase, a proposal for changes is obtained, mainly in the accessibility and usability of the application on mobile devices, as well as in the execution and correction of the specific visual exercises incorporated in the prototype. This phase lasts four weeks.

### WKS4: advanced technical capabilities

The last workshop is attended by domain experts and developers. A definition of the AI capabilities applicable to the prototype is made. As discussed above, recommender systems are designed to present the user with the appropriate item from a predefined set of items based on certain characteristics. These characteristics can be based on the items to be recommended themselves (content-based), on user behavior (collaborative filtering) or on a mixture of both approaches (hybrid methods). The recommender focuses on proposing an order of the exercises and the time to perform them, according to the indications of the visual rehabilitator and attending to the patients’ feedback. In this research work, the designed application allows the visual rehabilitator to decide the set of exercises that will be available to the patient. If we let the patient choose the exercises to be performed, it could happen that he/she does not choose the best option for his/her rehabilitation. It could be the case that the exercises chosen are either too easy for the user, making the rehabilitation a boring task to complete, or too difficult, frustrating the user. The result of this workshop is the identification of the features and attributes necessary for the future implementation of the recommender. This iteration is spread over two weeks.

## Results

### Functional lines, app features and activities (WKS1)

In the first workshop, the specific objectives proposed by the domain experts are developed, summarizing the association between user profiles and functionality, as well as the technological capabilities that the application must have. Finally, the activities to be implemented in the application are also defined on paper.

The specific objectives provided by the domain experts are summarized as follows:

- Develop an interface where practitioners and patients can access information on the patient’s progress in each exercise.
- To implement accessibility criteria adapted to the characteristics and needs of end users.
- Enable the monitoring, by patient and practitioner, of the evolution of the visual loss or the increase of the scotoma.
- Introduce the localization parameters of the peripheral retinal area to be rehabilitated in each patient and the system adjusts the presentation of the stimuli in each exercise.
- Provide a program of visual rehabilitation exercises for people with AMD, adapted to their visual loss and needs, with increasing levels of difficulty and with evaluation mechanisms to monitor and adjust progress.

#### General functionality and technological capabilities

Table 2 shows the general functionalities (FUs) defined by the experts for the two user profiles that have been defined, i.e., for people with visual impairment and for visual rehabilitators. In the case of visually impaired people, with a typical age range of 60-65 years, the possibility of identifying themselves or registering (FU1), performing rehabilitation exercises (FU2), being able to access their own medical record as well as the evolution of the visual loss (FU3) and being able to contact the visual rehabilitator (FU4) will be offered. Visual rehabilitators will be able to register as such (FU5), manage their account (FU6), manage patients’ accounts (FU7) and supervise the exercises performed by their patients (FU8).

**Table 2:** Software product functional lines by user profiles

	Functionality (FU)
Patient	FU1 - Register and login process
	FU2 - Rehabilitation exercises
	FU3 - Access medical record and evolution
	FU4 - Contact to visual Rehabilitator
Visual rehabilitator	FU5 - Register and login process
	FU6 - Manage my account
	FU7 - Manage patient account
	FU8 - Supervise patient exercises & evolution

The technological capabilities defined in workshop 1 are listed in Table 3, which summarizes the characteristics that the application must provide, divided into 3 blocks:

1. ** Mobile App (A1, A2 and A3):** Regarding the application interface, it must be accessible from a web browser (A1) and a mobile app developed for this purpose (A2). Access to the application will be through user accounts: practitioner and Patient. Each practitioner account is associated with its patients, while each Patient account is associated with a single practitioner. The practitioner will create the patients’ accounts by entering their access data and their visual status. The practitioner will be able to edit the data of his associated patients, as well as give them access to each of the exercises and adjust the parameters to adapt them to the therapeutic needs of each case. The database will store the conditions of use and the result of each test, that is, the location entered by the practitioner to place the stimuli in the activity, the specific activity parameters, and the patient’s progress with the exercises performed. Technical requirements: Only for devices with Android operating system and with a screen larger than 10X10 cm. Regarding the storage of the data tracking (A3), two situations are differentiated: (1) access from the mobile App and (2) access from the web interface. In case of access from the mobile App, the data will be stored on the user’s local device and periodically uploaded to a server with authentication. This makes it possible to work in environments with poor coverage or even without internet connection, although the user will be alerted that a network connection is necessary for the specialist to monitor the patient’s progress. In the case of access via the web interface, the data will be stored directly on the server.
2. **Accessibility (A4):** To improve the usability of the application by the elderly and visually impaired all elements of the application allow screen readers and tools such as Android Talkback can be activated to aid navigation. The patient design should have large, separate buttons, high contrast colors and large fonts that should allow for resizing.
3. **AI-based recommender (A5 and A6): **The first step before starting rehabilitation is to perform a diagnostic test to measure the extent of the patient’s visual degeneration, which will be performed by the health specialist and is independent of this application. Once the affected areas of vision have been identified, the specialist specifies the parameters and location of the peripheral retinal area to be trained, entering this information into the application to be considered during the rehabilitation (A5). The app has a test based on the Amsler grid through which the patient and the rehabilitator can monitor the evolution of the visual loss periodically (self-assessment) since the system stores the results of each test. The activities presented to the user must be managed by an AI system, which will ensure that the exercises are adapted to the specific needs of the patient and provide feedback on the progress achieved in each of the exercises performed.

**Table 3:** Profiles and their role in the collaborative project

Technical capability	Description
Mobile app	A1 - Web Interface
	A2 - Mobile interface
	A3 - Local and remote storage
Accessibility	A4 - Accessible design
AI-based recommender	A5- Adaptation
	A6 - Feedback on progress of patient exercises

#### Self-assessment and paper-based exercises

**Fig. 3:**
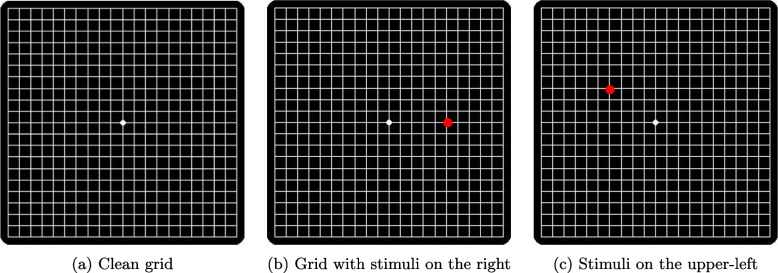
Amsler grids for self-assessment with presentation of stimuli to delimit the scotoma

**Requirements for the Amsler grid test:** This test allows to know the areas of visual sensitivity loss (scotomas) and to locate them in the user’s visual field to plan rehabilitation by the practitioner and to monitor changes and progression of visual loss. Devices with screens large enough to allow full display of the grid should be used. It is a 10cm x 10cm grid composed of parallel, vertical, and horizontal lines spaced 5 mm apart, and a central point (Fig. 3a). Each square subtends an angle of 1$^{\circ }$ and the entire grid 20$^{\circ }$ at a distance of 30 cm. To perform the test with each eye, the patient should be 30 cm away and should also maintain this distance to perform the exercises. It is important to maintain the recommended working distance in the exercises in order to achieve the desired results. This is also the case with classical rehabilitation. In our tests a cord attached to the edge of the screen was used, however, in the continuation of the project it is planned to control the distance using a computer vision system via the front camera of the device.

The grid test will be performed by showing 50 stimuli in the form of red dots, (for 3 seconds in the initial prototype, thus leaving 3 seconds to respond until the next stimulus appears) in different areas of the grid to check if the patient sees them, while maintaining fixation on the central point, situated parallel to the grid and at a constant distance of 30 cm, see Fig. 3b-c.

The test can be repeated periodically, and the results are saved in the database. Only the practitioner can delete failed results.

**Parameters and location of the treatment area:** The parameters and location of the peripheral retinal area to be trained will be entered by the practitioner manually in the form of coordinates or by marking them on the grid, so that the stimuli are presented in that area with respect to the fixation point. These parameters can be modified, only by the practitioner, according to the evolution of the disease or rehabilitation. The records of the grid tests and the location of the area to be rehabilitated will be saved in the database.

**Requirements for exercises:** they will vary, progressively increasing in difficulty. Statistics will show graphs of successful and unsuccessful results for each exercise. The fixation point may be increased in size to help patients maintain stable fixation. The exercises will consist of recognizing shapes, letters and objects appearing on the screen, projected on the retinal area selected for rehabilitation with respect to the fixation point, according to the coordinates entered by the practitioner. The center of the fixation point is considered to be the center of the 10x10 cm conceptual grid. The distance and location of the stimuli presented in each exercise are set with coordinates in relation to the center. If the width of the device is less than 10 cm, the position and size of the stimuli does not vary but, to maintain their projection on the same area of the retina that the practitioner has established, the user must always keep the working distance constant at 30 cm.

**Initial exercises:** In all the exercises the time between the presentation of the stimuli can be modified, depending on the needs of the patient on the screen and the start of each one. In all cases, the Edit Text is already filled with the default recommended time and the user can increase or decrease it according to his needs. It has error correction: in case the user deletes the default, time and does not fill in the new time before starting the exercise, the default time will be set automatically.

Each exercise is preceded by a start screen with instructions. Before starting each exercise, the patient must fix the red dot and tap anywhere on the screen when successful to start the exercise. If the patient wants to stop the exercise, he/she can tap on the top left of the screen and will return to the exercise start screen. At the end of each exercise the result with the number of correct and incorrect answers and the number of attempts is saved in the database and will be displayed on the final screen to the user so that he/she can decide to repeat the same exercise, modifying or not the parameters or return to the exercise menu and select the next one. The Talkback function is available in all exercises, both in explanation and execution. The action of touching a button will require a double click if Talkback is enabled and a single click otherwise. All texts are readable by touching them only once.

**Fig. 4:**
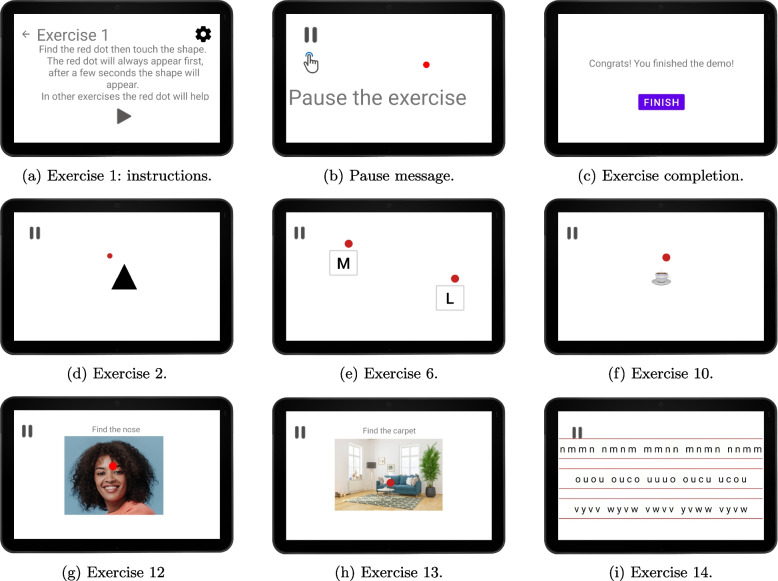
Exercises demonstration (**g** Photograph obtained from freepik.com)

Initially, 15 exercises designed by the visual rehabilitator were proposed as a starting point for the two end users to evaluate and suggest improvements. The proposed exercises were as follows:

- **Exercise 1:** demonstration of the operation with explanations so that the user can understand and test the way to interact in the following exercises. In (Fig. 4a) we can see precise instructions for carrying out the exercise using a large font. While performing the exercise, the user is informed that it can be paused at any time (Fig. 4b). Finally, upon completion of the exercise, the user will be informed (Fig. 4c).
- **Exercises 2 to 5:** In these exercises, different geometric figures appear in a random sequence. The user, keeping his gaze fixed on the red dot, must touch the figure that appears only if it matches the one shown at the beginning of the exercise. The complexity of the exercise increases according to the type of figure, from the most easily recognizable, such as simple solid figures (Fig. 4d) through hollow figures, bold letters and non-bold letters of various sizes.
- **Exercises from 6 to 9:** As in the previous exercises, the aim is to identify the requested image (a letter or a figure, as the case may be) that will appear in the treatment area. However, in this case the difficulty is increased, since the user is asked to alternate the fixation between two points, not to keep it in only one point, as in exercises 2 to 5. At the beginning of the exercise the user is asked to fixate the gaze on one of the red points of the (Fig. 4e), the one on the right of the screen, for example, and when he/she has achieved it, to touch the screen to indicate to the system that the exercise can begin. At that moment figures start to appear (in the same area with respect to the red dot as in the previous exercises since it is the area that has been set for the treatment). While the user looks at the point on the right, random images will appear, as in exercises 2 to 5, and when the image to be identified appears, the user must touch it and immediately fix the gaze on the point on the left to perform the same task. This alternation will be repeated until the end of the exercise.
- **Exercise 10:** a single fixation point is presented again, in this case with images of real objects, specifically drinks, see (Fig. 4f), and the user must tap on the requested image when they identify it.
- **Exercises 11 and 12:** the image of a face is presented, of a woman in exercise 11 and a man in 12, see (Fig. 4g), in which the fixation point, marked with a red point as before, changes position and once fixed, the user is asked to touch a specific part of the face.
- **Exercise 13:** the image of a room with furniture is presented (Fig. 4h). The red dot changes position and, once fixed, the user is asked to touch the location of a specific object in the room.
- **Exercises 14 and 15:** three lines of letters are presented, and, without the fixation point, the user has to tap on the letters requested in each case (Fig. 4i).

### Prototype testing by experts (WKS2)

**Fig. 5:**
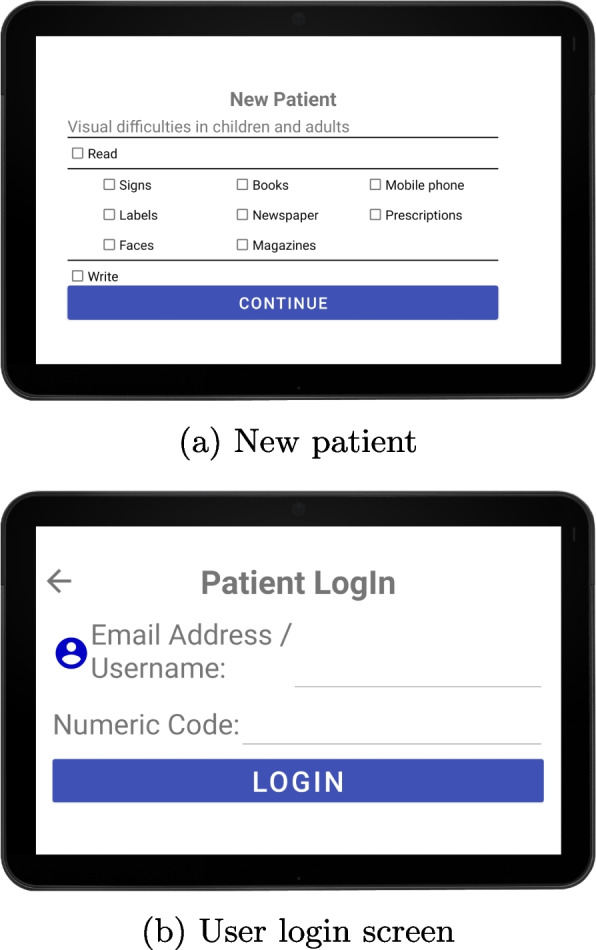
New user registration and login

During this phase of the project, a basic prototype is available with minimal functionality to test the interface, such as the self-assessment test using the Amsler grid, as well as a form for the rehabilitator to enter the parameters and location of the treatment area and the patient’s vision-related difficulties, such as difficulties in reading text, using a smartphone, recognizing faces, etc. (Fig. 5a).

Once the rehabilitator has entered this information, the application will generate a unique numeric code that can be sent to the patient so that he/she can register in the application (see Fig. 5b).

A tentative implementation of the exercises defined in the WKS1 is also available, which allows early identification of possible implementation and usability errors by the domain experts.

As a result of this phase, a beta version of the complete application is obtained, in which all the functionalities described in (Table 2) as well as the technological capabilities described in (Table 3) have been implemented.

### User testing of the application(WKS3)

As a result of this work meeting, the technical team has an understanding and a description of the required modifications to the initial prototype.

#### Proposals for changes

**Fig. 6:**
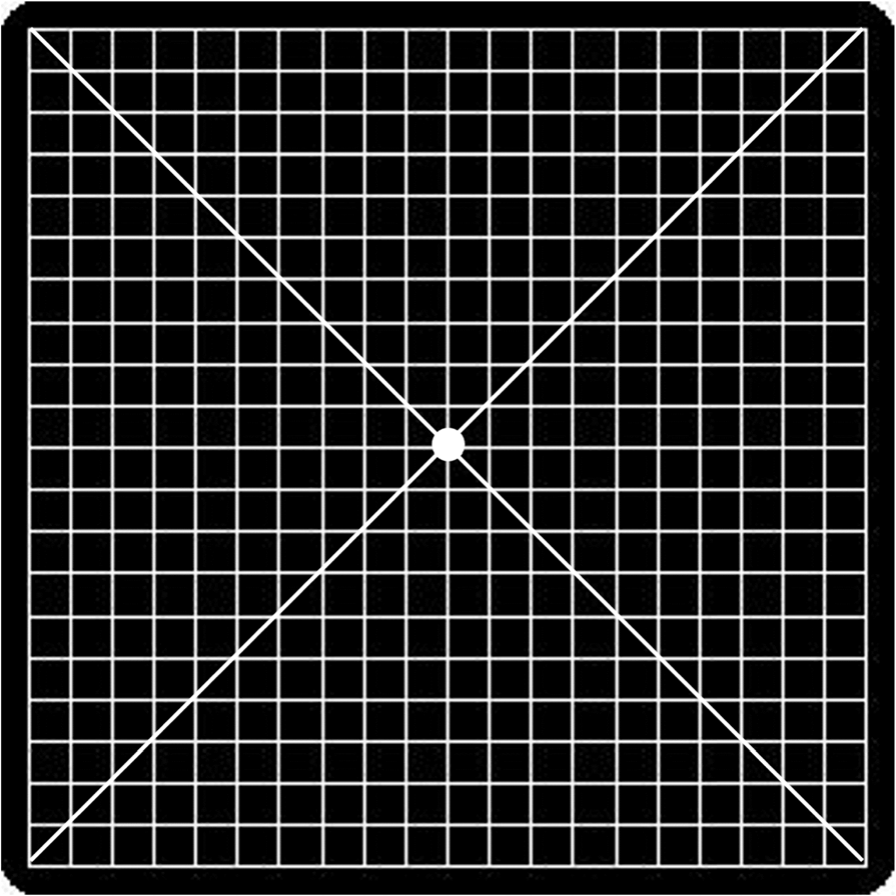
Amsler grid with diagonal lines and thicker center point

After evaluation of the initial application and its performance by the visual rehabilitator and patient users and following their proposals, the following changes were induced:

- **Interface:** The initial aesthetics were modernized.
- **Initial start-up screen:** The parameters and the location of the menus were adjusted to make it more user-friendly.
- **Handling:** Initial parameters were modified by increasing the font size.
- **Devices:** The grid test requires a screen of a minimum size that allows the full grid to be presented, but the execution in the exercises does not. Therefore, mobile devices can be used to perform the exercises.
- **Amsler grid:** The grid was replaced by the version with diagonal lines and a thicker center point to make it easier to locate the center (Fig. 6). In addition, in follow-up cases, the total number of stimuli can be modified by halving the number of stimuli presented to shorten the test, as well as limiting the area to be evaluated to the environment of the scotoma. Thus, the test can be reduced from 5 minutes to 2-3 minutes. This is very important considering the characteristics of the users. These parameters will be adjusted by the rehabilitator.
- **Exercises:** The practitioner will indicate to the patient whether to perform the exercises monocularly or binocularly and the system must be able to record how the exercises have been done which will self-adjust, reducing the difficulty, in the event that the negative results exceed the positive ones so as not to generate frustration for the patient. In all the exercises the practitioner can modify the time between the presentation of two consecutive stimuli, as well as the number of stimuli presented, their size and the duration of the exercise, according to the patient’s needs. This can only be configured by the practitioner, the patient user will not have access to these changes as they make the application difficult to use. The Talkback function will be disabled during the execution of the exercises, since having it activated can generate confusion with the dynamics of the exercises, since it is necessary to touch the screen to respond to the stimuli. The changes introduced in each of the exercises were as follows:
  - Exercise 1: Removed from the list of exercises and renamed “demonstration”.
  - Exercises 2 to 5 and 10: were modified so that the user did not have to touch specifically on the figure, as this introduced a greater complication to the exercise. When recognizing the image, the user simply has to touch anywhere on the screen. The time between presentations was also reduced from the default 10 seconds to 5 seconds, to make the test shorter.
  - Exercises 6 to 9: in these exercises, the images associated with the two fixation points appeared at the same time. In the new version, the behavior was modified to give the user time to change the fixation between one point and the other. In the new version, the user must fixate on the first point and then touch the screen to indicate that he/she is ready to start the exercise. At that moment, the figures associated with this fixation point will start to appear. If the user does not correctly detect the specified figure, he/she will be prompted to switch to the other fixation point. All this process will be guided with voice instructions in all cases to improve usability.
  - Exercise 10: the requested image must appear at least twice, and the default size has been reduced to make the exercise more difficult.
  - Exercises 11 and 12: were replaced by a single exercise with the same dynamics as exercise 10. It must be fixed on the red dot and faces appear in the eccentric vision zone, the user must touch the screen when the image of the requested face appears.
  - Exercise 13: it was decided that the red dot would not change position so that the user would know where it was and could locate it more easily in an area of the screen located outside the view of the room, and the image of the object to be searched for would appear next to the fixation point, in the position of the eccentric retinal area trained in all the exercises. It was made compulsory for the test to be voice-guided, even if the option was not selected.
  - Exercises 14 and 15 were not selected as they were considered too difficult for the users.

### Recommender system (WKS4)

Once a revised implementation of the visual rehabilitation application is defined, a recommender system is presented. The recommendation algorithm is proposed based on the work [45] as an automatic exercise selection loop. It is a content-based recommendation algorithm, as each exercise is modeled with a list of skills needed to complete the exercise, designed with the visual rehabilitator and attending to the patient.

The automatic selection loop starts choosing the next recommended exercise. The exercise is executed, and the results are stored. Afterwards, the user model is updated, and the patient can choose to continue performing his rehabilitation.

For this purpose, the following attributes are chosen to be considered within the recommender system we propose here:

- Hits and misses in the responses to the exercises.
- Patient’s reaction time and thresholds/tendencies to the appearance of each image in the exercises.
- Patient’s age, and other factors such as visual impairments.
- Compliance with each patient’s exercise program.

These attributes will be predefined in the application by the visual rehabilitator and based on the patient’s conditions and characteristics, different recommendations can be built. For example, if the number of successful exercises is low, the system should recommend easier exercises based on the user’s ability scores and exercise requirements. Alternatively, if a patient performs the tests with a high number of successes, the system should recommend a harder exercise, creating a new incentive and challenge for the user to improve himself. The recommendation system in this work has been designed under a content-based approach, as a closed-loop control system around exercise difficulty, in an effort to keep it slightly above the patient’s current ability level. For this reason, the difficulty of the exercises should be specified by the visual rehabilitator when generating the patient’s chart, as the exercises will not have the same difficulty for all patients, they will be designed for them.

## Discussion

This paper presents a pilot project aimed at designing a solution called Rehab-AMD. It is currently estimated that AMD affects 200 million people, and this number will increase significantly in the coming years [8]. This is why it becomes especially important to develop devices that improve the prognosis of those affected and are truly useful through the teamwork of software engineers, visual rehabilitation experts and people with AMD. Recent studies show that the co-design of digital applications in the health sector benefits greatly from the realization of working sessions (Workshops) with the participation of patient representatives and health specialists [20]. Following this premise, the pilot project incorporated the execution of specific and collaborative workshops between the domain experts and the technical team. Each iteration of the design process was based on a workshop, with a total of four workshops, which were preceded by an initial planning and participant identification session.

Conducting these workshops allowed for fluid communication and direct interaction between the domain experts and the technical team, which facilitated the understanding of the needs and perspectives of AMD patients. In addition, these workshops provided the opportunity to receive valuable feedback that contributes to the continuous improvement of the Rehab-AMD solution.

In our experience, the patient’s short-term motivation comes from their progress in solving more difficult exercises and improvement in their daily tasks. The greatest motivation is that progress in rehabilitation translates into greater ease in performing daily activities. Rehabilitation, being affordable and convenient from home, adapted to the patient’s progress and with professional supervision, reduces frustration. This increases the likelihood that the patient will continue with rehabilitation, which is crucial in the long term to manage the disease and minimise its impact on the patient’s life. Recent work, [46], has studied the difficulties encountered in the healthcare sector and the incorporation of users to identify barriers to the use of ehealth applications by patients. Among others, self-efficacy barriers, previous habits and negative image of novelty are identified. Working approaches such as the one shown in this project can help to overcome these barriers by involving users in the software design itself and by early participation in prototype testing and examples of use.

The pilot project described in this scientific paper highlights the importance of close and continuous collaboration between domain experts, user community representatives and technical software development teams. Co-design based on iterative and collaborative design principles, together with the conduct of specific workshops, ensures greater effectiveness in requirements definition, validation and acceptance testing of the Rehab-AMD solution for the visual rehabilitation, monitoring and information management of AMD patients. The result is a technological solution that meets the needs expressed by both patients and healthcare specialists. In addition, accessibility criteria have been taken into account to ensure ease of use by people over 60 years of age as end users. This work can serve as an example to other researchers and developers looking for practical and effective technological solutions for end users.

Of particular importance in this project is the coordination work between the different members of the group. During the integration of technological capabilities, the visually impaired and the age range of users identified in the analysis of user profiles have been taken into account [47–49], as well as the need for adaptation of the tool to the evolution of the patient’s disease. For this reason, coordination among the different members of the group was considered essential so that all participants would understand their role and any doubts that arose during project implementation could be effectively clarified. In this case, this work was carried out by two people, a software engineer, and a visual rehabilitator, who clarified questions related to pathology and visual rehabilitation to the software experts and the technical possibilities for patients and health practitioners. It also highlights the excellent willingness on the part of patients who, according to this experience, are eager to have their opinion and needs considered in the design of solutions they are intended to use.

To achieve the gradual adaptation of the technology to the patient’s evolution, the use of Artificial Intelligence (AI) models is also required. The AI helps to monitor compliance with the progress of the exercise program assigned to each patient, alerting the visual rehabilitator in case of non-compliance. The AI also should recommend the most appropriate training exercises for each patient based on their progress. It also performs the adaptation of the complexity and exercise program. This includes increasing or decreasing the level of complexity of the exercises depending on the reaction time and response (adjustment of the threshold of each patient) to the response pattern of each patient. At the same time, the application should be usable in both Web and mobile interfaces, to increase its availability to patients, incorporating design elements to ensure accessibility for the elderly, both visual and auditory.

## Limitations

The main limitation of this project is the small number of health experts and patients: one vision therapist and two patients. The lack of funding makes it difficult to involve more professionals. However, the commitment and experience of the current team compensates for this limitation. A small team was chosen to ensure better coordination and that patients felt the importance of their input. Two patients were considered sufficient to provide the most relevant information. In future phases, it is hoped to incorporate more patients and visual rehabilitation experts.

This project has focused on the design of the Rehab-AMD solution and its full implementation remains for future projects, incorporating the recommender system in future implementations, for its evaluation by a large group of end users. We believe that the co-design method used makes it much more viable for the solution to be useful for its purpose, in this case to improve visual rehabilitation, monitoring and information management of patients with AMD.

## Conclusions

Co-design in the Rehab-AMD solution is confirmed as a methodology that allows usability issues and needs to be identified by the user from the initial stages of design. This allows software developers to work on the certainty that the final result will be truly useful and manageable by the people with AMD and the targeted vision rehabilitators.

## Data Availability

This work does not have a set of statistical data to contribute. It is an application design process whose steps are detailed in the manuscript and whose result is a prototype app that must be conveniently implemented for future usability studies. This paper only presents the design.

## Abbreviations

AI: Artificial intelligence
AMD: Age-related Macular Degeneration
DE: Domain experts
FU: functionality
PRL: Preferred Retinal Locus
WKS: Workshops
